# Implementing Strengths-Based Dialogue to Reframe Clinical Education and Community Engagement

**DOI:** 10.1044/2023_PERSP-23-00018

**Published:** 2023-08-25

**Authors:** Monique T. Mills, Magdalen Balz, Daniel Price

**Affiliations:** a University of Houston, TX; b Boston University, MA; c University of Massachusetts Boston

## Abstract

**Purpose:**

Health care professionals want to solve problems. When health disparities are observed, the solution often rests on expanding access to clinical services. However, what are the varied paths that persons with communication disorders might take to access speech, language, and hearing care? Where are these paths successful and where does a well-intended initiative have an absent or limited effect in altering disparities? Multiple, complex factors affect access to health care in underserved communities. However, current practice tends to frame the goals and metrics of outreach programs in terms of access to health care services, which risks privileging the perspective of the providers who want to increase the volume of services accessed over the voices of the community members for whom access to health care is only part of the larger course of their lives. Solutions that do not reflect those community strengths outside the service provision framework likely yield minimal impact on quality of life, since the community members are less likely to fully embrace the solution.

**Method:**

In this clinical focus, we describe a community-informed strengths-based framework for clinicians and clinical researchers whose work is designed to reach underserved communities by employing mutual trust, empathy, active listening, and patient-centered care planning. Through case scenarios, we exemplify key tenets of the framework.

**Conclusions:**

The community-informed strengths-based framework detailed in this clinical focus supports a paradigm shift from a biomedically-informed strengths-based framework to a model of health care service provision that focuses on individual or community strengths. Eliciting guidance from those receiving care and framing the totality of encounters in terms of the process of responding to community strengths can build a collaborative and sustainable path forward toward achieving health goals.

Clinical education forms the foundation for professional development for graduate students in the fields of speech-language pathology and audiology. Effective clinical education “ensures that new clinicians are well prepared and that individuals with communication disorders receive quality services” (American Speech-Language-Hearing Association [ASHA], n.d.). Clinical educators help graduate students “develop professional behaviors, including the ability to work with individuals and their families” within the context of interprofessional education and practice (ASHA, n.d.). One of the most widely used methods of clinical education is the case-based learning scenario (ASHA, n.d.). Analyzing and discussing client cases provides a functional context for graduate students to learn about clinical decision making with a person-centered approach (Santana et al., 2018). Case studies foster learning that will generalize outside of the classroom to clinical work in the community (O’Fallon & Garcia, 2023). By reflecting on the specific needs of individual clients, students can learn about the impact of health care and educational policy and infrastructure on clinical service provision, as well as systemic inequities within health care.

Clinical education is incomplete without a focus on community health. In recent years, evaluation and treatment in speech-language pathology has become increasingly focused on person-centered care. However, the individual strengths of a client and their family are often measured without acknowledging a person’s surrounding community. Although community health initiatives are within the scope of practice for speech-language pathologists (SLPs; ASHA, 2016), there is limited guidance about how to implement community considerations in care planning, clinical research, and clinical education. It is becoming clear to clinicians, clinical educators, administration, and leadership that we are not optimizing health outcomes (Di Sante & Potvin, 2022; Wilson, 2019). Clinicians have recognized the need to move beyond cultural competence toward cultural humility (Gregory, 2020; Hall & Johnson, 2020). Yet, we are working within the constraints of our respective systems. Nevertheless, we need to move toward community engagement and dialogue (Gray, 2022). Reframing clinical education curricula to include strengths-based dialogue has the potential to position community engagement at the center of clinical work for the future.

Community health is traditionally framed by a strengths-based biomedical model in which individual community members take responsibility for their health by accessing health care experts as the need arises. Health care professionals, educated within the same model, then see themselves as servicing people with needs. There is an increasing awareness of the missed opportunity to embrace client strengths necessary to achieve health outcomes, such as patient activation scores, which measure a client’s engagement with their own health outcomes (Hibbard et al., 2004; Koh et al., 2013; Pekonen et al., 2020). Unfortunately, viewing clients as either needy or empowered runs the risk of reinforcing the biomedical frame that privileges the clinician’s activities against the community framing in terms of broader strengths. A focus on authentic dialogues between clients and clinicians, in which communication partners say and do what they genuinely believe, builds mutual trust necessary for optimizing health outcomes and forefronts the relationship between them, instead of highlighting the point of interaction where the service is exchanged (and “access” is realized). This clinical focus proposes a strengths-based frame, situated in the social determinants of health, in which individuals partner with health care agents to leverage their shared capacity for enhancing behavioral change through mutual trust. The innovation of a community-informed strengths-based framing is to understand the capacity as shared and co-created in the context of authentic relations among interested parties and evolving dialogues between client and clinician. Put another way, strength is shared more equally between client and clinician within a community-informed strengths-based model of health care. Table 1 illustrates key differences between a biomedically-informed strengths-based model and a community-informed strengths-based model of health. We explain the two frameworks in the sections that follow.

## From the Biomedical Model to the Social Determinants of Health

The biomedical model has been criticized for creating a sense of the body as a mechanism where disease is represented as an external aberration best treated by medical intervention, which devalues the individual’s agency and the holistic functioning of the body (Engel, 1960). Later criticisms turned to the systemic manifestations of the biomedical model and to the ways in which the neoliberal ideal of individual responsibility prevented researchers from seeing the holistic functioning of the socio-ecological context (Krieger, 1994). The slip in the criticism of the biomedical model is important for understanding how talk about the social determinants of health can remain tied to parts of a reductionist and disempowering model: If the final determination of health or illness is situated in the individual’s body, then the strengths-based approach is only about supporting the individual client’s capacity. Even when taken as a whole and as “determined” by social forces, the many ways in which the strengths of a community can shape the experience of health—for example, support or shame, accommodation or exclusion, trust or disengagement—are obscured by an approach that situates the responsibility for health in the individual client’s separate strength (Metzl & Hansen, 2014). A shared dialogue begins from a place of connection and not from an analytic separation. The tools associated with the biomedical model can be brought to bear within that shared dialogue, and the health care professional can support a shared sense of strength that acknowledges the community’s role without obscuring the embodied experience of the individual. The social determinants of health are then considered as part of the context for an effective shared dialogue—the clinician must understand what obstacles, goals, and strengths the client has—and not as an expert-driven explanation for the poor health outcomes of disempowered groups (Weinstein et al., 2017).

In some ways, this movement from a biomedical model to a shared-dialogue view of the social determinants of health represents a subtle change in emphasis in the way that one engages with clients, and yet it also explains why so many of the assessment protocols tend to perpetuate status quo or dominant epistemologies given that a priori assumptions are made without input from the tested population. The “nothing about us without us” movement is a call to take seriously the experiences and perspectives of our clients and research participants (Charlton, 1998). The commitment to a community-engaged and strengths-based dialogue shows us how to begin that process by critically appraising the biomedical model and the analytic tools that biomedical research has provided (Xie, 2021).

## Competence: From Cultural to Structural

Clinicians and their clients participate in structures and systems that facilitate or block progress to health and well-being. Cultural competence models assume that if we fix the clinician, then we would move toward meaningful, authentic, honest dialogue that builds mutual trust for a strengths-based problem-solving approach. Adjusting our lens so that we appreciate cultural difference and its impact on human behavior is important: We do not want cultural incompetence. Yet, cultural competence models miss everything that happens to the client outside of the clinical space, as the client navigates their own self-efficacy and their capacity to adhere to clinical recommendations while traversing complex health care systems. Professionals are more effective when they embrace cultural humility, which appreciates individual differences within and across cultural groups, as the intersectionality of one’s identity to provide a step toward a community-informed strengths-based model (Tervalon & Murray-Garcia, 1998). The culturally humble clinician and client would be less prone to stereotype or essentialize others. Yet, cultural humility may miss the impact of structural gaps and barriers on our ability to be healthy and well. For example, a client seeking nutritional advice for managing diabetes from a culturally humble clinician who provides a safe environment will still experience challenges of transportation to a place to exercise, residing in a food desert, and a limited budget to purchase healthy foods. A structural competence model would solve this problem by bringing community strengths explicitly into the shared dialogue developed between clinician and client. That is, the clinician in this case might, with appropriate cultural humility, recommend that the client with diabetes gather with others in their community to walk for 30 min each evening. However, the successful clinician has moved beyond cultural competence and humility into active problem solving with the client that marshals their situated knowledge—when she knows, for example, that there is a social worker creating a new support group to help navigate structural gaps or barriers in a shared context.

## Recruiting Representative Communities to Programming Embedded in Representative Communities

Clinical researchers who view participants as “subjects” may only reach out to or engage in dialogue with community members when it is time to recruit them into research projects. They take a transactional approach with community members that defies trust building. This practice aligns with a biomedical strengths-based model in that it focuses on a part rather than a whole. That is, research participants are simply a part of the clinical researcher’s agenda, rather than a co-driver of the entire research enterprise. As implementation science becomes central to how research studies are carried out, clinical researchers are beginning to align more with a community-informed strengths-based model by creating community advisory boards that provide opportunities for ongoing dialogue (McGaffigan, 2011). There is a need to move from consulting with community members and clients to posing questions together and involving them in everything from co-curricular course design to research design.

## Learning: From Initiation–Response–Evaluation to Project Based

The two models of health are akin to differences in how teachers interact with their students. Teachers often pose a question or explain a concept, allow students to respond, and then evaluate student’s responses within the initiation–response–evaluation (IRE) mode of questioning (Sinclair et al., 1975). The IRE default is also enacted by clinicians seeking to check that what they have said to clients has been understood. Indeed, knowing the steps one needs to take to complete evaluation or treatment is important and does offer strength to the student or client. However, the IRE model of questioning fails to leverage the strengths, ideas, and strategies that students and clients can offer. For example, teachers who engage their students in project-based learning follow the student’s interests as they scaffold students into higher levels of discourse about the topic of interest (Helm & Katz, 2016). Likewise, clinicians who deploy ethnographic interviewing (Westby et al., 2003) to find out how their clients experience the world may be more likely to discover resources that the client possesses to exert strength and resolve their communication needs than are clinicians who complete traditional medical intake forms. Clinicians who engage in in-depth interviewing will obtain a dynamic rather than static portrait of the client’s life.

## Communicating: Jargon to Plain Language to Dialogue

Moving toward a community-informed strengths-based model of health care requires switching from jargon to plain language in our spoken and written interactions with clients. Jargon refers to specialized vocabulary within a content area or profession (Swann, 2019). Jargon is part of the cultural milieu for the clinician. However, the client may leave the interaction without comprehension after listening to a clinician explain in jargon. To improve how well clinical messages are conveyed, clinicians must reduce jargon and increase plain language (Stableford & Mettger, 2007). Plain language refers to “grammatically correct language that includes complete sentence structure and accurate word usage” (National Institutes of Health, 2010). Plain language allows the clinician’s message to be better understood by clients. Yet, plain language fails to address structural barriers. For example, a client who reads a housing brochure may be overwhelmed and confused by the legalese and detailed application process. They can connect with someone who explains the form in plain language. Now, the client understands what they need to do, but they are held back because of a structural barrier, such as transportation needs or difficulty obtaining required financial documentation, to complete the application.

Only in dialogue do clinicians learn of the limiting structural barriers. A clinician’s well-intended treatment recommendations may be clearly articulated and explained, but impossible for the client to implement. For example, clinicians regularly invite clients to “come back next week” for a follow-up visit. This practice is easier said than done for many clients who face structural barriers, such as needing to take multiple city buses to visit a provider’s office or being unable to easily leave their own place of (self) employment for a midday appointment. Finding childcare to attend multiple clinical visits is also a very real structural challenge for persons across the socioeconomic spectrum.

## Solving Problems: Teach-Back Method to Active Listening and Solution-Focused Brief Therapy Philosophy

The teach-back method is designed to ensure understanding of health care instructions and recommendations for follow through after discharge from the inpatient setting or an outpatient appointment (Talevski et al., 2020). This client education model was designed to ensure accurate understanding and communication in a health care setting, and there is emerging evidence to show the efficacy of this strategy (Ha Dinh et al., 2016). Consistent with the biomedical-informed strengths-based model, this method focuses on accurate delivery and reception of information but does not explicitly include listening to a client/caregiver and identifying goals of care that elevate the strengths of the person and their community. In a community-informed strengths-based approach, the client is seen as a partner in care planning and a member of the health team and a plan of care first starts with deep, active listening (Luterman, 2017). After active listening, a provider can personalize a care plan that incorporates the strengths of the individual client and opportunities for support within their community network. In a solution-focused brief therapy approach, the clinician and client design goals collaboratively with a focus on person-centered solutions to achieve increased quality of life. The clinician provides positive reinforcement by identifying specific examples of success where the client leveraged their strengths together with the strengths from their community.

## Summary

Each of the movements described above from a biomedical strengths-based model toward a community-informed strengths-based model allow for regular, ongoing dialogue between health care professionals and clients, which can establish and maintain mutual trust. Clinical educators play a key role in shaping how graduate students help clients navigate structural barriers that prevent optimal health outcomes. We now turn to a pragmatic exploration of practical strategies for that transition with an emphasis on incorporating these concepts into clinical education.

### Case Scenarios

Case scenarios have been the foundation of clinical education for implementing evidence-based practice. They provide an important point of discussion and critical thinking (O’Fallon & Garcia, 2023). We present three clinical scenarios that illustrate the opportunities for dialogue that encourages strength at various points of clinical engagement in the field of communication sciences and disorders. Each is based on plausible interactions from actual field experiences, but no real identities are being described or disclosed. In the first, we present a case of child language assessment in which outcomes vary as a function of having established mutual trust and shared expectations. The second scenario describes a preventative intervention program to minimize cognitive decline among underserved older adults through social engagement and cognitive stimulation. Lastly, we present a model of the community health worker (CHW) as a potential advocate for productive dialogue between clients and clinicians. In each clinical scenario, there are multiple opportunities to exert mutual strength to meet communicative needs. We begin each case scenario with a list of the most salient model features to be drawn from the account.

## Case 1: Mobile Screening for Communication Disorders in Children

- Social determinants of health
- Programming embedded in representative communities
- Project-based learning
- Dialogue
- Active Listening and Solution-Focused Brief Therapy philosophy

A professor of communication sciences and disorders and her undergraduate students are conducting free hearing and language screenings on their department’s mobile unit at a back-to-school fair held each year by the city’s mayor. Annabelle, a chiropractor who is concerned about her son’s communication, is also at the fair to provide free spinal screenings. She thought about calling her husband to advise him to bring their son to the fair for the language screenings because she is concerned that his language may be delayed: Her son is approaching the age of 5 years and is having difficulty answering complex questions. Despite his apparent delays in communication, Annabelle feels her son is bright and worries that if she brings her concern to school personnel, they will “label him” and “hold him back” academically. She reads a *New York Times* article (McWhorter, 2022) opining on how widely used tests of language tend to stigmatize Black children like her son who sometimes speaks “Ebonics” (see also Ball & Bernhardt, 2008; Pearce & Williams, 2013). She is also aware that implicit and racial bias were significantly related to “patient–provider interactions, treatment decisions, treatment adherence, and patient health outcomes” (Hall et al., 2015, p. e60).

When she went home from the fair and talked things over with her husband, he empathized, but worried more about not getting their son the help he needed and suggested that they contact his pediatrician for advice. The pediatrician agrees with Annabelle’s observation and writes a referral for SLP services. Annabelle insists that the pediatrician provide them with a list of SLPs, preferably African American. Her husband called everyone on the list but, due to the covid-19 pandemic, was placed on several waiting lists. The SLP who was available and willing to come to their home for testing was an energetic young blonde woman with 2 years of experience. Annabelle was dubious, but her husband insisted that they move ahead with scheduling testing.

The SLP arrived a bit early so that she could create a RIOT. The RIOT assessment process entails Reviewing, Interviewing, Observing, and Testing (Langdon, 2002). She *r*eviewed the child’s medical history before the meeting and noticed that he was born within a geriatric pregnancy. She *i*nterviewed the parents to learn more about the language practices and routines in their home and about the child’s interests. The SLP’s concerns about her son’s interests and in trying to understand when and how he communicates left a positive impression with Annabelle that continued as the SLP *o*bserved her son playing with his toys, intermittently sprinkling in comments (e.g., “That’s a neat puzzle!; I wonder what piece goes there.”). Annabelle was relieved that the SLP interacted with her son very easily and naturally, as if they were old friends. The SLP then joined the child at his child-sized table for *t*esting. She started with a hearing screening, which the child passed. She moved on to a test of intelligence so that she could rule out developmental disability; the child performed within normal limits. The SLP then administered the Diagnostic Evaluation of Language Variation–Screening Test (Seymour et al., 2003), which determined that the child presented with some variation from mainstream American English (he was bidialectal) and was classified at the highest risk for language impairment. Finally, she audio-recorded Annabelle and her son engaging in free-play with his toys to transcribe and analyze later. Before she left, the SLP had coffee with the parents to offer some preliminary results that aligned with Annabelle’s observations, scheduled a follow-up visit for additional testing, and to plan for intervention goals, with input from the parents.

## Case 2: Minimizing Cognitive Decline in an Older Adult Who Experienced Unstable Housing

- Social determinants of health
- Project-based learning
- Programming embedded in representative communities
- Active Listening and Solution-Focused Brief Therapy

Jane is a 60-year-old woman with a chronic heart condition. She spent her adult years living paycheck-to-paycheck while raising her children, who are now grown and live several hours away. During a recent hospital admission followed by an extended inpatient rehabilitation stay, Jane missed several weeks of work and was then laid off. When she returned to her apartment, she was behind on rent, utilities, credit card payments, and several other bills. In the months that followed, Jane attempted to find a new job, but her health was not stable, and it was difficult to find employment that would work around her medical appointments. Her job search was ultimately not successful. She missed several months of rent and was evicted from her apartment. Like many older adults in the Boston area, she had difficulty finding an affordable apartment (City of Boston, 2022). Unfortunately, she did not have family or friends with room for her to stay or resources to support her financially. She also needed affordable housing that would be accessible with her wheelchair, and this further limited her housing options (Brown et al., 2017). Jane lived in transitional housing for several months. A few months ago, she was offered permanent housing through a local nonprofit organization. Since moving into permanent housing, she has attended medical appointments consistently and received assistance from staff for mail-order delivery of her medications.

Since being laid off from work, Jane lacked vocational fulfillment. She missed her daily interactions with new, young employees at work, where she would “teach them about life.” These interactions made her feel “important.” The program manager at her new residence recommended she join the Intergenerational Dialogue and Collaboration for Cognitive Wellness group (CogWell) that met weekly in her building’s dining room. She was initially reluctant, stating, “I don’t want to work on my memory right now.” She also felt her memory “hadn’t been the same” since the hospitalization, but she was reluctant to attend a group with memory drills where she might experience failure. She also felt lonely. The previous year of her life had been stressful, and she felt isolated without contact from her former coworkers and neighbors, as is often the case for unhoused persons (Cornwell & Waite, 2009; Levasseur et al., 2010). After learning that her new neighbor was attending CogWell, she decided to try the program.

Upon entering the dining room, Jane was given a name tag and welcomed by an SLP graduate student, who invited her to sit at an open table. The graduate student participated in two orientation sessions prior to starting this clinical experience. In these meetings, she and her classmates read articles about the high prevalence of cognitive impairment among people who are unhoused or experience homelessness (Stone et al., 2019). They also read and discussed articles about implicit biases and homelessness in the United States (Slayton, 2021; Terui & Hsieh, 2016). Prior to meeting the older adults in CogWell, students acknowledged their own implicit biases about homelessness and reflected on the potential effect of these biases during clinical work. Students aim to reshape implicit biases across the course of the semester through weekly conversations with faculty and classmates.

Although feeling vulnerable in her new role as clinician, the graduate student was prepared to engage with older adults in this supervised clinical experience. After sharing a bit about herself and the purpose of the program, the graduate student asked Jane about her favorite pastime activities. With hesitation, Jane shared that she had been extremely tired recently, so she spent most of her time watching television. The more time she spent alone, the harder it felt to initiate interactions with others. With support from her clinical supervisor, the graduate student acknowledged Jane’s feelings of loneliness, validated her courage to try group today, and then asked her about her favorite television show. Jane listed some of her favorite sitcoms from the past few decades. Unfamiliar with these titles, the graduate student found clips on YouTube and watched them with Jane. With enthusiasm, Jane described the context and drama among the characters. After the two reminisced about Jane’s favorite episodes, the graduate student engaged Jane in a conversation about her hopes and goals for the future using solution-focused brief therapy (BRIEF, 2016; Burns, 2006). Through a series of open-ended questions, Jane explained that, although she dreamed about going back to her old job, she would feel successful and accomplished if she could find a way to “feel like a leader again.” Jane explained that taking on a leadership role would make her valuable to her new community. Jane and the graduate clinician planned to take a turn leading the CogWell group in a subsequent week by eliciting favorite shows from the larger group and watching YouTube clips at the suggestion of the other residents and graduate students. Jane and the graduate student successfully facilitated a dynamic group conversation with rich, reminiscing memories and laughter. Feeling a sense of purpose, Jane became a regular member of CogWell and invited other residents to join.

In the subsequent weeks, Jane shared with the other residents and graduate clinicians that the instability in her life was fueled by losing her job and then her housing. Moving into stable housing, having a case manager, and joining the CogWell group all improved Jane’s outlook on the future, her access to health, and her feelings of connectedness. It did not, however, resolve all her challenges. In addition to cognitive decline and loneliness during her time of housing instability, she also experienced physical decline. She told the CogWell group that she was “supposed to be exercising more.” When a graduate student asked a follow-up question, Jane responded, “Oh we don’t really need to talk about it; that’s all I wanted to say for now.” This interaction highlights the importance of ongoing case management to support Jane’s complex medical needs. Stable housing alone or access to one medical provider is not sufficient to help most older adults like Jane. However, stable housing and communicating with providers, who are guided by a case manager, can change the trajectory of Jane’s health and her quality of life.

## Case 3: Optimizing Wellness Outcomes Through CHWs

- Social determinants of health
- Structural competence
- Programming embedded in representative communities
- Dialogue

The work of CHWs has recently been broadly celebrated as a key element in integrating vulnerable populations into their own care (Kim et al., 2021; Zulu & Perry, 2021). CHWs have found a wide range of roles in health care, including providing coordination, navigation, psychosocial support, chronic disease management, and a wide variety of health education and outreach. The fundamental idea at the University of Houston Community Health Workers Initiative is to approach care coordination as individual health advocacy oriented by a shared sense that the health care and social service systems are failing, and not the individuals who are having difficulty accessing appropriate care. The CHWs work with the individuals to create plans that navigate the difficulties with the system and allow them to optimize their moments of contact with the health system and to achieve their personal health goals.

For example, a CHW recently made first contact with a family at a local food distribution site and had a series of care coordination meetings with Cai, the mother of the family. The CHW’s role in food distribution was to ensure that every client of the food distribution nonprofit had optimal access to the broader health care system, based in an understanding of the family’s own goals and barriers faced. It soon became clear that Cai was not able to thrive because childcare was so difficult to secure. Childcare is difficult for many people in her community, and she had resigned herself to not being able to address the issues. However, as the CHW entered into deeper dialogue with the family, it became clear that the most difficult child to care for had signs of a developmental language disorder, which Cai had seen but not felt equipped to address. She was simply too overwhelmed with other priorities and fatalistic about the situation. How could she spend even more time on one child, when access to childcare was already such a burden? The CHW working with Cai, however, had training in resource utilization for families and in the types of health resources available at the university. She understood that appropriate engagement with the university’s speech and hearing clinic could actually reduce the overall burden. Because of the connection back to the university’s expertise, the CHW was able to arrange appropriate screening for the child and to begin the application process for extra social service support for the child. Although the process is ongoing, that support should then make other goals achievable for the family, including the childcare arrangements for the family as a whole. Finally, the successful health advocacy makes it more likely for Cai to trust the CHW on other health messaging and reinforces the sense that she and her family are a source of strength and self-determination in the process, with the appropriate help from the social service sectors and the health care professionals. The fatalism about the situation can be replaced with a measured optimism and resilient sense of long-term engagement with appropriate experts in a responsive health care system.

Although some CHWs are employed as front-office staff or play the role of case manager for clients and families, the CHW in this case scenario was not employed by the university’s speech and hearing clinic and was not directly involved in the delivery of speech-language pathology services for the child. The CHW engagement with Cai and her family points to a different role, parallel to the difference between interventions focused on cultural competencies and those that address structural determinants of access. The CHW in the clinic makes it easier and more comfortable to be in the clinic and to succeed in those interactions. The CHW that is fully embedded in the community ensures that the clinic is an option. In Cai’s case, the CHW was able to make concrete recommendations for creating better overall outcomes because the training in available services included SLPs in the university clinic.

In this case, the relationship is established as a partnership through the dialogue between the CHW and Cai, as oriented by the developing understanding of the paths that Cai is already on and the ways that they can better navigate the available services. For SLPs, the relationship with clients is always mediated by the referral process, but that fact does not preclude the opportunity to enter into authentic and meaningful dialogue with clients and with the CHWs who are helping them navigate the health care system. CHWs can serve guest lecturers in graduate courses to provide clinical educators and graduate students with authentic accounts of structural barriers to successful care and the importance of listening to complete and nuanced accounts of the community context for care. CHWs can help create better conditions for dialogues between individual clients and SLPs by grounding both sides of the conversation in concrete experiences of long-term goals, specific barriers, and the variety of strategies required to navigate the structural challenges faced by both clients and clinicians.

## Conclusions

Clinical education should advance solutions to health care problems. To solve health care problems, health care professionals must go further than cultural competence. Indeed, there has already been a shift in much of the health care community from cultural competence to cultural humility, but this shift is only a precondition to authentic shared dialogue. Recent calls have been made to increase cultural responsivity in SLP practice to mitigate against reproducing inequality and bias on the basis of dialect in the United States (Farrugia-Bernard, 2018). Although the critical move beyond cultural competence to cultural humility is underway (humility is better than hubris), a yet further step is to move into authentic dialogue with clients to assess and address cooperatively the structural barriers that confront clients from all walks of life and that beset health care providers. As evidenced in Case Scenario 2, authentic and meaningful dialogue does not immediately resolve all health disparities. Rather, dialogue establishes a sure space to progress toward wellness.

Policy change is important but insufficient to address structural barriers. There is a gap between what policy documents say and the actual practices within the health profession (Pascoe et al., 2018). In no way are we suggesting that policy changes are unimportant. Yet, we recognize that to make a meaningful impact on structural gaps and barriers, an open dialogue in identifying and leveraging community strengths is critical for immediate change. This task is easier for frontline professionals such as physicians and CHWs. For professionals such as SLPs and audiologists, who are not in frontline contact with the community, it is more complicated to see the structures that hinder authentic dialogue and to respond to structural gaps and barriers with their clients: SLPs and audiologists must unwrap “patients” who are referred to them by teachers and physicians, to see them as persons.

Moreover, the expectation that university clinics must serve as a revenue source may prevent them from serving as a resource to support the solutions arrived at through authentic dialogue. This entrepreneurial model also forces university researchers to decide to focus their time, talents, and resources into winning grants rather than building community relationships required to mend structural gaps and barriers. The move to structural understanding allows SLPs to see themselves as working with clients and other professionals, such as CHWs, to address the broader range of challenges faced by the clients and to ensure the best outcomes for the process.

## Figures and Tables

**Table 1.** Shifting toward a community-informed strengths-based model of health care service provision.

Biomedically-informed strengths-based model	to	Community-informed strengths-based model
Biomedical model	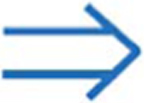	Social determinants of health
Cultural competence	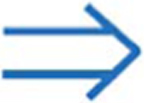	Structural competence
Recruiting representative communities	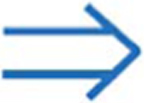	Programming embedded in representative communities
Initiation–response–evaluation	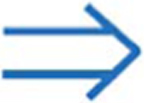	Project-based learning
Jargon and plain language	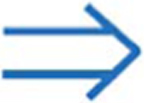	Dialogue
Teach-back method	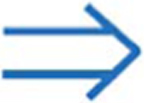	Active Listening and Solution-Focused Brief Therapy

## Data Availability

No data sets were generated or analyzed during this study.
